# Prevalence of selected genomic deletions and duplications in a French–Canadian population-based sample of newborns

**DOI:** 10.1002/mgg3.12

**Published:** 2013-05-21

**Authors:** Tracy Tucker, Sylvie Giroux, Valérie Clément, Sylvie Langlois, Jan M Friedman, François Rousseau

**Affiliations:** 1 Department of Medical Genetics, University of British ColumbiaVancouver, British Columbia, Canada; 2 Centre de Recherche du CHU de Québec-Hôpital St-François d'AssiseQuébec, Québec City, Canada; 3 Child and Family Research InstituteVancouver, British Columbia, Canada; 4 Department of Molecular Biology, Medical Biochemistry, and Pathology, Université LavalQuébec, Québec City, Canada

**Keywords:** 16p11.2, 22q11.2, normal population, screening

## Abstract

Chromosomal microarray analysis has identified many novel microdeletions or microduplications that produce neurodevelopmental disorders with a recognizable clinical phenotype and that are not observed in normal individuals. However, imbalance of other genomic regions is associated with a variable phenotype with intellectual disability (ID) or autism in some individuals but are also observed in completely normal individuals. Several large studies have reported the prevalence of copy number (CN) variants in people with particular features (e.g., ID, autism, schizophrenia, or epilepsy); few studies have investigated the prevalence of genomic CN changes in the general population. We used a high-throughput method to screen 6813 consecutive cord blood samples from a predominantly French–Canadian population to assess genomic CN in five genomic regions: 1p36, 15q11-q13, 16p11.2, 16p11.2-p12.2, and 22q11.2. We identified one deletion and one duplication within 1p36, two deletions of 15q11-q13, eight deletions of 16p11.2-p12.2, two deletions and five duplications of 16p11.2, and six duplications of 22q11.2. This study provides estimates of the frequency of CN variants in an unselected population. Our findings have important implications for genetic counseling.

## Introduction

In the last decade, chromosomal microarray (CMA) has improved our ability to detect and study genomic copy number (CN) changes. CMA has identified many novel microdeletion/microduplication syndromes associated with intellectual disability (ID) or autism that were previously unrecognized by karyotype analysis. Many of these pathogenic CN changes have a recognizable clinical phenotype and are not seen in normal individuals. However, other recurrent genomic microdeletions/microduplications have been reported in patients with ID or autism of varying severity, sometimes in association with additional phenotypic features and may also occur in completely normal individuals. Five regions in which CN changes are associated with variable phenotypes are discussed below.

Individuals with deletions within 1p36 (MIM 607872) present with hypotonia and ID, which is severe to profound in the majority of cases. In a study of 134 patients with 1p36 deletions, a significant proportion presented with additional features including microcephaly, large anterior fontanelle, and dysmorphic features (deep-set eyes, midface hypoplasia, flat nasal bridge, and pointed chin), although few patients presented with a common constellation of features (Gajecka et al. 2007). In addition, seizures, hearing loss, cardiomyopathy, or structural heart defects were frequently seen (Gajecka et al. 2007). Large deletions of this region have not been reported in normal individuals.

Deletions of 15q11-q13 cause Prader-Willi syndrome (PWS; MIM 176270) or Angelman syndrome (AS; MIM 105830), depending on the parental origin of the deletion. The phenotype of these disorders is well established, and deletions of this region have not been reported in individuals without ID or autism (Cooper et al. 2011). In contrast, duplication of 15q11-q13 (MIM 608636) is often associated with autism (de Vries et al. 2005; Christian et al. 2008) or psychotic illness (Consortium International 2008) but has also been reported in normal individuals (Schroer et al. 1998; Consortium International 2008). Studies of patients with autism (Schroer et al. 1998) or psychotic illness (Ingason et al. 2011) have found that the duplication is more often inherited from the mother or derived from the maternal chromosome and individuals who have a duplication on the paternal chromosome are less likely to present with a phenotype, suggesting a parent of origin effect with the duplication as seen with the deletion.

The phenotype associated with a deletion of 16p11.2-p12.2 (MIM 613604) has only been described in a few case reports (Ballif et al. 2007a; Battaglia et al. 2009; Hempel et al. 2009). The del16p11.2-p12.2 patients all have ID and expressive language difficulties and variable combinations of facial dysmorphisms, including downslanting palpebral fissures, deep-set eyes, and low-set-posteriorly-rotated ears. These features are nonspecific and make it difficult to associate them unequivocally to this CN change.

There has been only one case report of three patients (twins and one unrelated individual) with the reciprocal duplication of 16p11.2-p12.2 identified by CMA (Tabet et al. 2012) and four others identified by fluorescent in-situ hybridization (FISH) analysis, which are assumed to be the reciprocal duplication but cannot be confirmed as such (Engelen et al. 2002; Finelli et al. 2004). These seven patients all present with autism, and most had ID and language impairment, but no consistent facial features. In addition, four duplications of 16p11.2-p12.2 have been reported in DECIPHER (ID 263405, 250062, 888, 2131), but the phenotype was only described in one (ID 2131) and included developmental delay, dysmorphic features, and short stature. Deletions or duplications involving the common 16p11.2p12.2 breakpoints have not been reported in control individuals without ID or autism (Cooper et al. 2011).

Smaller deletions or duplications of 16p11.2 (MIM 611913; 614671) are ∼600 kb and can fall within the larger 16p11.2-p12.2 deletion region (Tabet et al. 2012). Deletions of 16p11.2 are associated with a variable phenotype, with patients presenting with autism or ID, and this small deletion is also observed in normal individuals (Weiss 2008; Bijlsma et al. 2009). In a large study, 16p11.2 deletion patients were noted to have dysmorphic facies with some having a similar facial appearance, although no characteristic facial features were noted that would suggest a recognizable syndrome (Bijlsma et al. 2009). Duplications of this region have been reported to be a risk factor for autism (Weiss 2008) but have also been reported within families with both affected and nonaffected individuals carrying the duplication (Weiss 2008). Moreover, deletions and duplications of this region were identified in studies of control individuals without ID or autism (Cooper et al. 2011).

Deletion of 22q11.2 is the cause of DiGeorge/Velo-cardio-facial syndrome (DG/VCF; MIM 188440/192430). This disorder is characterized by facial dysmorphisms, palatal clefting, or insufficiency and conotruncal heart abnormalities. In addition, individuals with this deletion may have ID, autism, or schizophrenia. In the majority of affected individuals, del22q11.2 occurs de novo; however, inherited deletions have been reported in 6–28% of cases from parents who were reported as phenotypically normal, although upon evaluation some features of 22q11.2 deletion syndrome are often noted (Wilson et al. 1992; Leana-Cox et al. 1996; Digilio et al. 1997, 2003; Thompson and Davies 1998; McDonald-McGinn et al. 2001).

The more recently recognized reciprocal 22q11.2 duplication (MIM 608363) causes a very diverse, nonspecific phenotype that may include ID, delayed psychomotor development, growth retardation, or hypotonia (Wentzel et al. 2008). Many of those affected have inherited the CN change from a phenotypically normal parent. The variable phenotype and high rate of inherited dup22q11.2 variants from phenotypically normal parents make defining the penetrance of this disorder difficult.

There have been several large studies of the frequency of CN variants in people with particular features (e.g., ID, autism, schizophrenia, or epilepsy); however, few studies have assessed the prevalence of CN changes in an unselected non-HapMap population (Botto et al. 2003; Cooper et al. 2011). This, in part, may be due to the cost of large-scale microarray studies, the sample size that would be needed, the need for CN validation by an alternate method and lack of access to large sets of population-based samples. As many of the CN alterations described above have a variable phenotype that is not very distinct and some have been reported in normal individuals, the aim of our study was to assess the incidence of these CN changes in an unselected population in order to compare the observed frequency with published incidence and address the question of penetrance. For this, we developed a multiplex ligation-dependent probe amplification (MLPA) assay to assess genomic CN within each of these five CN variable regions in 6977 unselected newborn cord blood samples, predominantly from a French–Canadian population.

## Materials and Methods

### Subjects

Cord blood samples that remained after routine biochemical and blood typing were collected from consecutive newborns at St-François d'Assise Hospital Obstetrics department in Quebec City between 1994 and 2004, and they were made anonymous and unlinked after tagging newborns from the same mother. The study was approved by the institutional ethical review board of Centre Hospitalier Universitaire de Québec. Patients admitted in the obstetrics department were mostly (93.3%) of French–Canadian descent according to a recent census (Santé-Québec 1992–1993). To preserve further anonymity of participants, we did not collect samples of 15% of all deliveries, randomly, thus making sure that any women having given birth in the institution during that time could not know whether her baby was part of the study or not. Only the sex of the baby was registered in the database.

### Preparation and quantification of DNA samples

Upon receipt of a cord blood sample, information on sex and familial relationship were entered into a database, and each sample was given a unique identifier and barcoded. A 200 μL aliquot of each sample was loaded on a 96-well plate. For each plate, six wells were kept empty for controls.

DNA was purified from the 200 μL blood sample in the 96-well plate format using the QIAamp blood kit (Qiagen, Mississauga, Ontario, Canada) according to the manufacturer's recommendations, and DNA was eluted in 200 μL of elution buffer. These served as the master plates. DNA quantification was performed with the Quant-it Picogreen dsDNA assay kit as described by the manufacturer (Cat# P7589, Molecular Probes, Invitrogen, Carlsbad, CA). The mean concentration was 43 ng/μL ± 19 (SD). Working plates containing 100 ng of DNA in a final volume of 10 μL per well were prepared from each plate.

### MLPA probe preparation

Probes for each locus were prepared as described by Schouten et al. (2002), with the exception that all probes were made synthetically (Eurofins MWG Operon, Huntsville, AL) as described previously (Stern et al. 2004). All probes were tested individually, and amplification was checked on a 2% agarose gel. Upon successful amplification of each probe, a mix of all probes was prepared, and DNA samples obtained from Coriell with known deletions or duplications (Table S2) were tested along with normal DNA. This step determined the concentration of each pair of probes and the polymerase chain reaction (PCR) conditions necessary for a multiplex assay.

### MLPA reaction

The MLPA assay was performed as previously described (Schouten et al. 2002) with some modifications. Briefly, DNA samples in working plates were split into two to perform duplicate reactions. In each plate, 5 μL of DNA sample containing 50 ng of DNA was heated at 98°C for 40 min. Samples were mixed with 1.5 μL salt solution (600 mmol/L KCl, 200 mmol/L Tris-HCl pH 8.3, 1 mmol/L EDTA) and 1.5 μL probe mix (containing 3 fmol of each probe, see Table S1), and heated at 95°C for 1 min followed by incubation at 60°C overnight. Four microliters of 10× Ampligase buffer and 1 unit Ampligase thermostable DNA ligase (InterScience, Ontario, Canada) were added to the samples to a final volume of 40 μL. Samples were incubated at 55°C for 15 min, followed by 5 min at 98°C. PCR amplification using 5 μL of ligation product was performed in a final volume of 30 μL containing 0.2 mmol/L dNTPs, 10× Qiagen HotStar buffer, 0.5 unit Qiagen HotStar Taq DNA polymerase, 6 pmol of unlabeled primer (5′-GTGCCAGCAAGATCCAATCTAGA-3′) and 6 pmol of labeled primer (6-FAM, VIC, NED, or PET 5′-GGGTTCCCTAAGGGTTGGA-3′). The differentially labeled products were mixed, and 0.5 μL was combined with 0.2 μL LIZ standard and 9.8 μL formamide and denatured for 2 min at 95°C. The products were sized on a 16-capillary 3130XL Genetic Analyser using POP-7™ polymer as matrix (LifeTechnologies, CA) and analyzed using GeneMapper® Software version 4.0. Data were exported as a text file for each plate for each color to perform calculations of CN for each probe.

### Calculation of CNs

A custom interactive Excel spreadsheet was developed to facilitate data handling and CN analysis of GeneMapper text files. CN calculations were performed using peak areas and peak heights independently, the results were compared and a final genotype was reported. The average area or height of the reference probes (Chromosome 7, Table S2) was calculated and used to normalize each probe to reduce variability. To determine CN, the normalized peaks for each sample were divided by the normalized peaks of, initially, two control samples with two copies of each probe run on the same plate. The calculation was first performed using these two known controls and more control individuals were added when the CN was clearly 2 for each probe. We found that using the maximum number of control samples gave more reliable results.

Each probe's CN was expressed as a dosage quotient, where a value of 1.0 indicated the presence of two alleles, lower values (<0.75) represented a deletion, and higher values (>1.3) represented a duplication. When there was discrepancy between the CN calculated using height and area, “ND” was entered as the probe ratio for both results. The result of the duplicate sample was compared automatically to that of the first sample to give a final genotype. When there was discrepancy between the duplicates, each result was manually inspected and for those reactions that were different between the duplicates, an additional MLPA reaction was performed. A total of 77 samples yielded ambiguous results and were reanalyzed in a third and fourth MLPA reaction.

### Sex determination by MLPA

An X chromosome probe was used to determine the sex of each individual and cross-referenced to that recorded in the database and to the results of a PCR reaction for a Y chromosome-specific sequence (5′-CCTTGCAATCTCTCTTAATGG-3′ and 5′-TCATGAAAGACACTTTGGACG-3′). For all discordant sex classifications observed, the Y chromosome-specific PCR was repeated along with a positive control (MCAD primers targeting medium-chain acyl-CoA dehydrogenase gene) as previously described (Giroux et al. 2007).

### Validation with TaqMan® CN assays

For every sample with a genotype of 1 or 3 copies, the MLPA result was confirmed using a TaqMan assay for the same region as the MLPA probes but using a different sequence (see Table S3). The assay was conducted essentially as described by the manufacturer (Applied Biosystems, Foster City, CA). Briefly, 10 ng of genomic DNA was tested in three or four replicates. Each reaction included 5 μL 2× TaqMan® genotyping master mix, 0.5 μL TaqMan copy number specific assay mix and 0.5 μL TaqMan copy number reference assay mix in a final volume of 10 μL. The reaction plates (fast optical 96-well reaction plate, Applied Biosystems) were sealed with optical adhesive film (Applied Biosystems) and run on a 7500 Fast Real-time PCR System (Applied Biosystems) at 95°C for 10 min and 40 cycles at 95°C for 15 sec and 60°C for 60 sec. Relative CN was calculated using the 2^−ΔΔCt^ method (Livak and Schmittgen 2001) with CopyCaller® software (Applied Biosystems).

### Statistics

We calculated the lower and upper limits of the 95% confidence interval using the Wilson procedure as described by Newcombe (1998). Calculations were performed using the free tool available at http://vassarstats.net/ and SAS version 9.3 (SAS Institute Inc., Cary, NC) for the Fisher exact test.

## Results

We selected CN variable regions 1p36, 15q11-q13, 16p11.2, 16p11.2-p12.2, and 22q11.2, each of which is CN sensitive and has been implicated in ID. In addition, patients with CN changes of these regions show variable phenotypes and some of these CN alterations have also been observed in normal individuals. Within each of these regions, we selected a gene in the minimal region of overlap or critical region to design one MLPA probe (Table S1). These genes are *GABRD* (1p36; MIM 137163), *GABRB3* (15q11-q13; MIM 137192), *PRKCB* (16p11.2-p12.2; MIM 176970), *TBX1* (22q11.2; MIM 60254), and *SEZ6L2* and *KCTD13* (16p11.2), *SEZ6L2* and *KCTD13* (MIM 608947) are adjacent genes, and their CN state was always concordant within this sample set.

We screened a total of 6977 unselected newborn cord blood DNA samples in duplicate by MLPA. The procedure used (see Materials and Methods) allows a single technician to run, analyze, and interpret a thousand samples in 1 week. In total, 164 samples were excluded; 99 samples failed to give a result for any probe in both MLPA reactions (1.42% of samples), 12 were excluded because the DNA concentration as measured by a Picogreen assay was too low (below 4 ng/μL) to give a reliable MLPA result (Schouten et al. 2002), and 53 were excluded because the blood sample was duplicated or not from a newborn.

In all, 6813 samples remained and were used to calculate the CN state frequencies for each locus. In total, we identified 11 deletions and 12 duplications (Table 1). We identified one deletion and one duplication within 1p36 and two deletions but no duplications of 15q11-q13. For the larger CN variable region, 16p11.2-p12.2, we identified eight deletions and no duplications, and for the smaller CN variable region of 16p11.2, we identified two deletions and five duplications. Finally, we did not identify any deletions but identified six duplications within 22q11.2.

**Table 1:** Summary of CN states in 6813 newborn cord blood samples for regions analyzed

CN state	1p36	15q11-q13	16p11.2		16p12	22q11.2
					
	*GABRD*	*GABRB3*	*SEZ6L2*	*KCTD13*	*PRKCB*	*TBX1*
0	0	0	0	0	0	0
1	1	2	2	2	8	0
2	6811	6810	6806	6806	6805	6807
3	1	0	5	5	0	6
ND	0	1	0	0	0	0
Total	6813	6813	6813	6813	6813	6813

CN, copy number; ND, not determined because no DNA left to repeat testing.

An X chromosome MLPA probe was also used to evaluate assay performance. Phenotypic sex was collected for each infant, and the phenotypic sex was confirmed by Y chromosome-specific PCR. In all, 6776 final sex genotypes were obtained after two MLPA reactions; the X probe failed for 37 samples that were not retested. Comparison with the sample database indicated 13 discordant results. Close inspection of each discordant result led us to recalculate ratios with only one reference probe for four plates because the ratios of four different individuals were low (between 0.75 and 0.90) for female and the database indicated a male. Reanalyzing the whole plate with only one reference probe recovered the four erroneous genotypes but did not change any other genotypes. Two MLPA results were rejected because they were in a gray zone between two and three copies, and the seven remaining discordant genotypes were clearly nonambiguous with respect to the MLPA result. For these seven discordant genotypes, we tested each sample with a Y chromosome-specific PCR and internal control (see Materials and Methods). In five cases, two X chromosomes were observed along with a positive Y chromosome PCR, consistent with Klinefelter syndrome, which has a prevalence of 2 in 1000 male fetuses (Bojesen et al. 2003; Herlihy et al. 2011). In addition, we identified two individuals with only one X chromosome by MLPA who were negative for the Y chromosome PCR, consistent with Turner syndrome, which has a prevalence of 1 in 2000–2500 liveborn females (Hook and Warburton 1983). Therefore, of these 13 discordant results, only six were likely to have been misgenotyped by the MLPA assay, giving 99.9% accuracy for this X chromosome probe.

### False positive and false negative rate

We included four controls in each plate: two with known deletions or duplications of one of the tested regions obtained from the Coriell Cell Repository, and two normal female individuals (two copies for each locus as determined by TaqMan assays). We compiled the raw data (peak area ratios only) for these four controls from all plates analyzed and calculated the number of false negatives for each probe (ratio >0.75 or <1.3 for deletions or duplications, respectively). To estimate the number of false positives, we calculated the number of individuals expected to have two copies (ratio between 0.75 and 1.3) who were found to have a ratio <0.75 or >1.3, indicative of a false positive deletion call or a false positive duplication call, respectively, after a single MLPA assay.

Thirteen percent of control individuals provided no result, leaving 570 control results available for analysis for most probes. With the numbers obtained we calculated the error rate with 95% confidence for the size of the sample tested (Table 2). We observed a 1.75% genotyping error rate for the X probe in the smaller sample tested after a single MLPA assay but the complete genotyping test (after comparing duplicate assays) indicated six erroneous genotypes out of 6776 calls, which corresponds to an error rate lower than 0.1% (0.04–0.2%, CI 95%). For the other probes, the error rate among the controls was lower, suggesting a similar low error rate in the entire sample (Table 2).

**Table 2:** Number of false negative and false positive results observed after a single MLPA reaction obtained with Coriell DNA and normal female DNA used as controls in each plate

	False negative/Total true positive	False positive/Total true negative	% error with 95% confidence interval
Probe X Sex	3/83	7/487	1.75 (0.95–3.2)
Probe *GABRD*	Not applicable	9/568	1.6 (0.8–3.0)
Probe *GABRB3*	2/93	2/466	0.7 (0.3–1.8)
Probe *SEZ6L2*	8/93	5/477	2.3 (1.3–3.8)
Probe *KCTD13*	1/93	8/477	1.6 (0.83–3.0)
Probe *PRKCB*	4/47	3/523	1.2 (0.6–2.5)
Probe *TBX1*	0/98	5/471	0.9 (0.4–2.0)
Total observed	18/507	39/3469	1.43 (1.1–1.8)
Overall% error with 95% confidence interval	3.5 (2.3–5.5)	1.1 (0.8–1.5)	

Each plate contained two Coriell cell DNAs and two normal female DNAs chosen randomly, and each plate was run twice in two independent reactions. On average, 570 genotypes were available for each probe. MLPA, multiplex ligation-dependent probe amplification.

While the number of false negatives and false positives seems important after a single MLPA assay, the fact that the reaction was performed twice allowed recovery of most genotypes. With the tool developed to compare duplicate MLPA results, no erroneous genotype among the controls was called, but discordant results between the two MLPA assays were tagged and identified for a second genotyping round if needed. The standard deviation for the calculated ratio was 8–11.5% for each probe, similar to previous reports for synthetic probes (Stern et al. 2004).

## Discussion

Despite progress made in the development of CMA technology, such testing is still relatively expensive, labor intensive, and difficult to adopt for high-throughput screening. In order to obtain accurate estimates of CN variant frequencies, previous studies have used quantitative PCR or TaqMan assays (Perry et al. 2007). However, such methods are also expensive and require multiple replicates to obtain reliable results. Mefford et al. (2009) have reported a rapid method to genotype rare CN changes in large sample sets using the Illumina GoldenGate SNP genotyping array with customized probes and an algorithm to provide automatic data analysis.

We developed a reliable MLPA procedure that is inexpensive and amenable to high-throughput screening with high specificity and sensitivity. We found that the technique could identify the sex of individuals with 99.9% accuracy using a single probe targeting the X chromosome. The analysis of controls with known genotypes and sex showed an estimated error rate of 1.75% (95% CI 0.95–3.2) for that probe after a single MLPA assay. We have confidence in the results of the autosomal probes as the estimated error rate from 570 tested control samples after a single MLPA assay was lower (0.7–2.3%, Table 2) than the X chromosome probes. In addition, discrepancies between duplicates of MLPA results were automatically flagged for a manual inspection and/or an additional MLPA reaction. Finally, all genotypes different from the expected two copies were tested with TaqMan to confirm the deletion or duplication.

In this study, we screened 6813 cord blood samples from unselected newborn infants for CN changes at five loci. Table 3 provides a summary of CN change frequencies of these loci in ID cases and controls from the literature.

**Table 3:** Summary of the reported pathogenic frequency, CN frequencies reported by Cooper et al. in ID cases and adult controls without ID and comparison to study findings for each of the five genomic regions tested

Region	CN state	Frequency of pathogenic CN change	Size of pathogenic CN change	Number reported in 15,767 ID cases Cooper et al. 2011(frequency)	Number reported in 8329 adults without ID Cooper et al. 2011 (frequency)	Number reported in current study(frequency)
1p36	Del	1 in 5000 (Heilstedt et al. 2003)	Variable, but up to 10 Mb (Heilstedt et al. 2003)	79 (0.005)	0	1 (0.00015)
1p36	Dup	Few case reports (Heilstedt et al. 1999) and 1 DECIPHER cases (ID#255469) with 2.3 Mb duplication inherited from normal parent	Variable, and often with concomitant genomic imbalance	16 (0.001)	1 (0.00012)	1 (0.00015)
15q11-q13	Del	1 in 10,000–15,000 (Cassidy and Driscoll 2009; Van Buggenhout and Fryns 2009)	5–7 Mb	16 (0.001)	0	2 (0.0003)
15q11-q13	Dup	1 in 30,000 (Battaglia 2008)	Variable	27 (0.0017)	0	0
16p11.2-p12.2	Del	Few case reports (Cooper et al. 2011; Tabet et al. 2012)	7–8 Mb	2 (0.00013)	0	8 (0.0012)
16p11.2-p12.2	Dup	Very few case reports (Cooper et al. 2011; Tabet et al. 2012)	7–8 Mb	2 (0.00013)	0	0
16p11.2	Del	Unclear (Weiss 2008; McCarthy et al. 2009; Crepel et al. 2011; Golzio et al. 2012)	600 kb	64 (0.004)	3 (0.0004)	2 (0.0003)
16p11.2	Dup	Unclear (Weiss 2008; McCarthy et al. 2009; Crepel et al. 2011; Golzio et al. 2012)	600 kb	28 (0.0018)	2 (0.0002)	5 (0.0007)
22q11.2	Del	1 in 2000 (Shprintzen 2008)	1.5–3 Mb	96 (0.006)	0	0
22q11.2	Dup	Unclear	1.5–3 Mb	50 (0.003)	5 (0.006)	6 (0.0009)

CN, copy number; ID, intellectual disability.

ID affects 1–3% of the population; therefore, of the 6813 infants screened, 68–204 would be expected to present with some form of ID. We observed 11 CN losses and 12 CN gains at five loci for which CN changes had previously been associated with ID, a variable phenotype and/or occurrence in normal individuals. With the exception of 1p36, the CN variability of the analyzed regions arises as a result of nonallelic homologous recombination (NAHR) between low copy repeats. This mechanism would be anticipated to produce equal numbers of deletions and duplications at each locus. On the basis of the reported frequencies of these CN variable regions in liveborn or control populations without ID or autism (Table 3), we expected to see ∼9 deletions and ∼9 duplications for all loci combined (details below). Overall, we observed a higher frequency of CN variants, but the difference is not statistically significant.

### 1p36 region

Deletion of 1p36 is one of the most common subtelomeric deletions (Heilstedt et al. 2003). The majority of cases (52%) have a terminal deletion, whereas in 29% of cases, the deletion is interstitial (Shaffer and Bejjani 2006; Ballif et al. 2007b). The 1p36 deletion is not mediated by repetitive elements within the region, and, therefore, no common breakpoints are observed. Instead, the deletion is thought to arise by premeiotic breakage-fusion-bridge cycles (Ballif et al. 2003).

In our series, we identified one *GABRD* deletion (prevalence = 0.00015, upper limit of 95% CI, 0.0008) consistent with the reported frequency of live births (0.0002). Given that deletions of this region have not been reported in normal individuals or to have been inherited from a normal parent (Table 3), the individual with 1p36 deletion we observed would be expected to have ID with some of the dysmorphic features observed in del1p36 patients (Gajecka et al. 2007). Because our samples were anonymized, we do not know whether this is true.

There are very few cases of dup1p36 reported without concomitant genomic imbalance of another region resulting from a chromosomal rearrangement. The small number of cases of 1p36 duplication reported could in part be due to the duplication having a milder phenotype than the corresponding deletion of this region or represent a benign variant, as duplications of *GABRD* (but not deletions) are reported in the Database of Genomic Variants (DGV).

In our series, we identified one *GABRD* duplication (upper limit of 95% CI of prevalence, 0.0008), consistent with the literature (Table 3). As the whole genome was not analyzed, it is not clear if this CN alteration was isolated, complex or the result of inheriting an unbalanced chromosomal rearrangement.

### 15q11-q13 region

Deletions of 15q11q13 that cause Prader–Willi syndrome or Angleman syndrome (PWS/AS) can range from 5 to 7 Mb and are mediated by NAHR between flanking low copy repeats, generating common breakpoints among patients.

In our series, we observed two deletions of the *GABRB3* gene (prevalence = 0.0003, with 95% CI = 0.0001–0.0011), which is within the expected range based on the frequency of PWS and AS syndrome (Table 3). Without studying the parents, it is unclear if the deletions are maternal or paternal in origin, which would determine the phenotype.

Duplications of the 15q11-q13 region can result from an interstitial duplication of 15q11-q13, the reciprocal product of the deletion. Alternatively, gain of CN can occur by a supernumerary chromosome formed by the inverted duplication of proximal 15q, known as isodicentric chromosome 15, resulting in tetrasomy for the 15q region. The majority of reported cases of dup15q11-q13 result from a supernumerary isodicentric 15 (Schroer et al. 1998). Overall, duplications of this region resulting in a phenotype are thought to occur in 1 in 30,000 live births (Battaglia 2008) (Table 3). We did not find any duplications of this region in our series, despite duplication (but not deletions) entries in the DGV.

### 16p11.2-p12.2 region

There are a number of low copy repeats within the 16p11.2 region, and at least two distinct rearrangements mediated by NAHR are reported. One rearrangement involving 16p11.2-p12.2 can span up to 7–8 Mb. All of the deletions and duplications of this region have a common distal breakpoint, but they differ in the proximal breakpoints due to the involvement of different low copy repeats in the NAHR event (Hempel et al. 2009). The second rearrangement within this region, 16p11.2 (16p11.2 discussed below), is ∼600 kb size and may fall within the 16p11.2-p12.2 region if the rearrangement involves the most proximal low copy repeat. However, in most reported cases, the proximal breakpoint does not include the 16p11.2 region.

We observed eight deletions of *PRKCB* in our series (prevalence = 0.0012, 95% CI = 0.0006–0.0023). This is the largest number of CN changes found for any of the regions analyzed and significantly larger than the 0.00013 frequency observed among patients with ID by Cooper et al. (2011) (Table 3). Interestingly, there are no entries for this gene (deletions or duplications) in the DGV. Moreover, 16p11.2-p12.2 is also the region with the fewest reported pathogenic CN changes. As there are so few reported cases of this deletion in patients with ID, it is likely that not all of the individuals identified in our study would present with an ID phenotype. As CN changes in this gene have not been reported in patients or controls (Table 3), we cannot conclude if the CN changes detected represent the common deletions mediated by NAHR or if they are a smaller benign polymorphism or smaller pathogenic mutations that are too small and are not well covered with current whole genome microrarrays.

CN changes within this region are thought to occur by NAHR (Tabet et al. 2012); therefore, an equal number of deletions and duplications is expected, as was observed in the Cooper et al. (2011) study (Table 3). In contrast, we did not observe any duplications of this region (upper boundary of the 95% CI = prevalence 0.0006). We observed significantly more deletions than duplications at this locus (Fisher exact test *P* = 0.0078), which is striking, considering that duplications are generally better tolerated than the corresponding deletions. The fact that fewer duplications of this region were observed could suggest selection against the duplication or alternatively, the deletion represents a common polymorphism in the French–Canadian population.

### 16p11.2 region

There have been a number of studies looking at the prevalence of 16p11.2 CN changes in ID and autism, and these CN changes appear to be enriched in cases versus controls (Weiss 2008). In our series of 6813, we identified two individuals with deletions (prevalence = 0.0003) of *SEZ6L2* and *KCTD13* (95% CI = 0.0001–0.0011) and five individuals with duplications (prevalence = 0.0007; 95% CI = 0.0003–0.0017). Both *SEZ6L2* and *KCTD13* have entries (deletions and duplications) in the DGV. The CN states observed for *SEZ6L2* and *KCTD13* were always concordant in the samples included in this study. On the basis of the frequencies reported in control populations, we would expect to observe at least two deletions and one to two duplications in our series (Cooper et al. 2011).

We observed more duplications than reported among controls in the Cooper study (Cooper et al. 2011), but the difference is not statistically significant. Interestingly, by targeting a smaller region by MLPA, which included *SEZ6L2* and *KCTD13*, Golzio et al. (2012) found six deletions (1.2%) and two duplications (0.4%) among 518 cases, a frequency significantly higher than reported by Cooper et al. and our series (Table 3).

Also, in our series, and in keeping with other reported cases, we did not identify any individuals with deletions or duplications involving all three of the genes (*SEZ6L2*, *KCTD13*, and *PRKCB)* targeted in our assays of 16p11.2 and the 16p11.2-p12.2, showing that the 16p11.2-p12.2 CN changes do not include the 16p11.2 commonly deleted region.

### 22q11.2 region

CN changes of 22q11.2 are recurrent, usually 1.5 or 3 Mb in size, and mediated by NAHR involving flanking low copy repeats (Lupski and Stankiewicz 2005). Deletions resulting in a DG/VCFS phenotype are thought to occur in 1 in 2000 individuals (Table 3) (Shprintzen 2008). Duplications of this region have only been recently identified as disease causing, and the frequency among ID cases has not yet been determined. Similar to the other CN changes mediated by NAHR, one would expect an equal number of deletions and duplications to occur. Both deletions and duplications of *TBX1* are reported in the DGV.

In our series of newborns, we did not observe any deletions of *TBX1*. On the basis of the reported frequency of phenotypic cases (Shprintzen 2008) (Cooper et al. 2011) and controls without ID (Cooper et al. 2011), we would have expected to see three cases among the 6813 newborns. The lower frequency of this deletion could be explained if pregnancies were terminated following a prenatal diagnosis made after the detection of a fetal cardiac defect. Alternatively, the prevalence of the disorder could be lower in this population than reported by Shprintzen (2008) or the sample population is too small and our observation is due to chance alone. We observed six individuals (frequency = 0.0009) with a duplication of the *TBX1* gene, similar to the frequency observed in a previous report (Table 3).

### Strengths, limitations, and clinical implications of this study

There are a number of limitations to this study. First, the sample size, although more than 6800, is too small to assess precisely the frequency of rare events in the general population. With this sample size, on the basis of the reported frequencies of some of the CN changes, we would expect to see at least one deletion of each CN variable region and 0–1 duplications for each region studied. As expected, we observed as many CNV duplications (12) as deletions (11) overall.

Although we observed a high number of deletions in 16p11.2-p12.2, there are no comparable normal population data available for comparison. We also observed large numbers of duplications in 16p11.2 and 22q11.2, significantly more than deletions of these same regions, but in order to determine whether this difference is statistically significant, a larger sample size is necessary.

Another limitation of this study is that only one probe was used for each locus (except 16p11.2), and therefore we cannot provide information concerning the size of the copy variations we observed or be certain that the CN change is not part of a complex rearrangement. The probe for the TaqMan assays targeted exactly the same region as the MLPA assay but different sequences. With the exception of the CN changes within 1p36, the majority of the CN variants reported in the regions studied have common breakpoints. While there are CN changes noted in single individuals in DECIPHER that do not have the common breakpoints (Table 3), the most likely scenario is that almost all of the variants observed in this study were mediated by NAHR with the common breakpoints. However, further study is necessary to determine whether this hypothesis is correct or whether we have identified benign polymorphisms that lie below the level of clinical CMA resolution.

In addition, parental samples were not available, and, therefore, we cannot determine whether the observed CN changes were de novo or inherited. Furthermore, these samples are anonymous and no follow-up information is available. Therefore, we are not able to determine whether these individuals with the CN changes have any of the symptoms that have been associated with genomic imbalance. Finally, the population studied was predominantly French–Canadian, therefore similar study in other populations is necessary to determine whether the observed CN variable frequencies that are discrepant from published literature represent a common variant in this population or reduced penetrance.

The study demonstrates the effective use of an inexpensive and reliable method (MLPA) to screen a large number of samples for specific CN changes. This method can be applied to larger population-based studies in the future to determine the frequency of CN variable regions that may be associated with abnormal phenotypes. Our method could be improved using more and longer synthetic probes in a single assay.

## Conclusions

In conclusion, the results of our study provide estimates of incidence for 10 clinically relevant CN changes in an unselected population-based sample of newborn infants in a French–Canadian population. We identified 11 deletions and 12 duplications in CN variable regions with known variable phenotype in 6813 unselected cord blood samples. This study highlights the need for additional larger studies involving unselected populations to better understand the overall incidence of these CN changes. In addition, longitudinal follow-up studies are needed to determine the clinical consequences of CNVs that are identified at birth (or prenatally) in the absence of phenotypic information.

## Supporting Information

**Table S1.** MLPA probe genomic location and sequence for each test and control locus studied.

**Table S2.** Description of cell lines used from Coriell.

**Table S3.** TaqMan® copy number assays used for validation.
