# Supplementary material for: Prevalence of selected genomic deletions and duplications in a French–Canadian population-based sample of newborns
Source: Mol Genet Genomic Med. 2013 May 21;1(2):87–97. doi: 10.1002/mgg3.12 (PMC3865573; doi:10.1002/mgg3.12)
Supplement: Supplementary file 1 [file mgg30001-0087-SD1.doc]

Supplementary Table 1: MLPA probe genomic location and sequence for each test and control locus studied.

| Gene | Chr | Genomic position (Hg19) | Left probe sequence | Right probe sequence | Size  (bp) |
| --- | --- | --- | --- | --- | --- |
| *GABRD* | 1p36 | Chr1:1956702 | 5’**GGGTTCCCTAAGGGTTGGA**ggCGGCAGGGTCCCATCGTGGCTCCCATGCTGGGCCGGCCT 3’ | 5’(P)CAGTCCTTCTTAGTTCTGCTCTTTCCTTGCAGGCCCCCCCGTGc**TCTAGATTGGATCTTGCTGGCAC** 3’ | 127 |
| *GABRB3* | 15q11q13 | Chr15:26866450 | 5’**GGGTTCCCTAAGGGTTGGA** GGCACACAGACCTGAGCCCATACAGCACTGTCCCA 3’ | 5'(P)TCAGGGTGAAGACGGATCATGCGGTTTTTCACTG**TCTAGATTGGATCTTGCTGGCAC** 3’ | 111 |
| *SEZ6L2* | 16p11.2 | Chr16:29882970 | 5’**GGGTTCCCTAAGGGTTGGA**GAATGAGGAGGGGAGGGGCGTCCTGGGTC 3’ | 5’(P)CTGCAGCTGTAGTCTTGGGGTTCAGATG**TCTAGATTGGATCTTGCTGGCAC**3’ | 99 |
| *KCTD13* | 16p11.2 | Chr16:29937100 | 5’**GGGTTCCCTAAGGGTTGGA**ctCGCTCGTACCTCCGGCATCGGTCAGCACCT 3’ | 5’(P)CCACGCGGCCGCTGAACATGGCTTTGAGC**TCTAGATTGGATCTTGCTGGCAC** 3’ | 103 |
| *PRKCB* | 16p12p13 | Chr16:24046799 | 5’**GGGTTCCCTAAGGGTTGGA**GACCACACGGAGCGCCGCGGCCGCATCTACATC 3’ | 5’(P)CAGGCCCACATCGACAGGGACGTCCTCATTGT**TCTAGATTGGATCTTGCTGGCAC** 3’ | 107 |
| *TBX1* | 22q11.2 | Chr22:19767261 | 5’**GGGTTCCCTAAGGGTTGGA**CTATGAGGATGATTCCCTCAAACTGGTTCTGAAAGCAGT 3’ | 5’(P)AGCTGTGCTTTTCTCTATCGCGTTCTTATTCTTTCTCCGACGT**CTAGATTGGATCTTGCTGGCAC** 3’ | 123 |
| *AIFM1* | Xq26 | ChrX:129265710 | 5’**GGGTTCCCTAAGGGTTGGA**GATGTTGGCTATGAAGCTATTGGTCTTGT 3' | 5’(P)GGACAGTAGTTTGCCCACAGTTGG**TCTAGATTGGATCTTGCTGGCAC** 3’ | 95 |
| ref115 | 7q31 | Chr7:114298300 | 5’**gggttccctaagggttgga**cacgaaggcgacattcagacaaatacaacattcc ca 3’ | 5’(P)tgtcatcaggtaggatatgaatgctcagtagagtttc**tctagattggatcttgctggcac 3’** | 115 |
| ref131 | 7q31 | Chr7:114270150 | **5’GGGTTCCCTAAGGGTTGGAt**ggcaagaatagtcttagatcttccta taacaaggct cttgctgtca 3' | 5’(P)aagttgcagtattatatatttttactgagcttagtaacagtg**TCTAGATTGGATCTTGCTGGCAC 3'** | 131 |

The sequence in bold is the common sequence complementary to the universal MLPA PCR primers.

Supplementary Table 2: Description of cell lines used from Coriell.

| Cell line | Description | sex | probe  X | probe  *GABRD* | probe  *GABRB3* | probe  *SEZ6L2* | probe  *KCTD13* | probe  *PRKCB* | probe  *TBX1* |
| --- | --- | --- | --- | --- | --- | --- | --- | --- | --- |
| Na05875 | del(16)(p11.1p11.2) | female | 2 | 2 | 2 | 1 | 1 | 2 | 2 |
| Na04435 | duplication chr16 and chr21 | male | 1 | 2 | 2 | 3 | 3 | 3 | 2 |
| Na09189 | del(15)(q11q13) | female | 2 | 2 | 1 | 2 | 2 | 2 | 2 |
| Na13325 | del(22)(q11q11) | female | 2 | 2 | 2 | 2 | 2 | 2 | 1 |
| Na03184 | 3 copies of chr15 | male | 1 | 2 | 3 | 2 | 2 | 2 | 2 |

Supplementary Table 3: TaqMan® Copy-number assays used for validation.

| Tested gene | Position Build 37.1 | TaqMan assay ID | TaqMan position |
| --- | --- | --- | --- |
| *GABRD* | chr1:1950768-1962192 | Hs00520782_cn | chr1:1956400 (exon 2) |
| *GABRB3* | chr15:26788693-27018927 | Hs01588442_cn | chr15:26866584 (exon 5) |
| *SEZ6L2* | chr16:29882480-29910580 | Hs01058805_cn | chr16:29883043 (exon 18) |
| *KCTD13* | chr16:29917660-29937545 | Hs00324778_cn | chr16:29937195 (exon 1) |
| *PRKCB* | chr16:23847300-24231932 | Hs01483855_cn | chr16:24046865 (exon 5) |
| *TBX1* | chr22:19744226-19771116 | Hs00278664_cn | chr22:19766764 (exon 10) |
